# Factors affecting HIV counselling and testing among Ethiopian women aged 15–49

**DOI:** 10.1186/s12879-019-4701-0

**Published:** 2019-12-21

**Authors:** Asfaw Negero Erena, Guanxin Shen, Ping Lei

**Affiliations:** 1 Department of Immunology, School of Basic Medicine, Tongji Medical College, Huazhong University of Science and Technology, Wuhan, China; 2 Madawalabu University, College of Medicine and Health Sciences, Bale Goba, Ethiopia

## Abstract

**Background:**

HIV voluntary counseling and testing (VCT) is a crucial gateway to all strategies related to care, prevention and treatment of human immunodeficiency virus (HIV) infection. Nevertheless, utilization of voluntary counselling and testing (VCT) service among adults is very low in Ethiopia. The objective of this study is to identify determinants associated with VCT utilization among adult women aged 15–49 in Ethiopia.

**Methods:**

A cross–sectional study was conducted based on data taken from the Ethiopian Demographic Health Survey (EDHS) 2016. Using cluster sampling, 14,369 women aged 15–49 years were selected from all the nine administrative regions and two city administrations. Logistic regression was used to analyze factors associated with HIV VCT utilization.

**Results:**

Overall prevalence of ever tested for HIV was 53% (95% CI, 52, 54). Aged 20–44, ever married, being at higher socio economic position (SEP) and having risky sexual behavior were factors which are positively associated with VCT utilization. Being Muslims in urban and protestants in rural were factors significantly and negatively associated with VCT utilization. Those who had stigmatizing attitude both in urban and rural and who had comprehensive knowledge in rural were less likely to utilize VCT service.

**Conclusion:**

VCT utilization among women in Ethiopia is demonstrating better improvement in recent years. However, stigmatizing attitude continued to be among the major factors, which are negatively affecting VCT uptake among women in Ethiopia. Concerted efforts should be made by all stakeholders to mitigate stigma, improve socio economic inequities and increase awareness on the benefit of VCT in controlling HIV in the society. In this aspect, the role of religious leader, schools, health extension workers and community leaders should not be undermined.

## Background

HIV testing is a cornerstone for all HIV prevention strategies and been promoted as an important first step strategy to detect, treat, and prevent human immunodeficiency virus (HIV) infection. But unfortunately more than half of the people living with HIV do not know their sero-status [1–3]. Successful HIV prevention is largely depending on improved HIV testing technology and HIV testing practice in the community. Hence, HIV voluntary counselling and testing (VCT) is a gateway to all systems of AIDS-related care [3, 4]. According to the United States Center for Disease Control and Prevention (CDC) recommendation, HIV screening should be done for all patients aged 13 to 64 years in all health care settings, including hospital emergency departments, urgent care clinics, inpatient services, sexually transmitted disease clinics, tuberculosis clinics, and primary care offices after informed consent is obtained [5, 6].

Legal, policy and social barriers continued to restrict individuals access to HIV care services and HIV testing remains infrequent regardless of its pivotal role in controlling HIV epidemic [7, 8]. Almost all countries are still far from realization of United Nation’s Joint program HIV/AIDS (UNAIDS) plan to end AIDS epidemic by 2030 and achieving 90% diagnosis for all HIV infected individuals by 2020. According to data analyzed from Demographic and Health survey of 15 sub-Saharan Africa, 23 to 71% of people living with HIV knows their sero-status. Similarly systematic data analysis of national HIV treatment from 69 countries showed that HIV diagnosis (target one—90% of all HIV-positive people diagnosed) varied from 87% (the Netherlands) to 11% in Yemen [9–11].

Even though women are at higher risk for HIV acquisition and the prevalence of HIV among adults in Sub-Saharan Africa is enormously high, women’s access to HIV testing is uneven and HIV testing and counseling uptake among women remains at its low level [12–14]. Especially adolescent girls and women bear disproportionate HIV burden accounting for two out of three new infection occurring each day globally [15].

Ethiopia has also been implementing HIV voluntary counselling and testing (VCT) as a key strategy in its effort to prevent and control HIV/AIDS in the country. However, the utilization of VCT service among males, females, adults and people living in rural areas of the country is still low [16, 17]. According to Ethiopia Federal Ministry of Health (2007) report, women are particularly vulnerable to HIV infection and face multiple challenges in making decisions concerning their reproductive lives. Therefore, counselling is an important tool to empower women to make informed decisions that would help them prevent HIV infection (Federal HIV/AIDS Prevention and Control Office Federal Ministry of Health July 2007).

Understanding determinant factors affecting utilization of VCT helps policy makers in an effort to design effective strategies toward preventing and control of HIV/ADIS including improving the uptake of VCT in general public and among women specifically. In Ethiopia information on factors determining HIV testing among women is not sufficiently available. The current study aimed at identifying underline factor preventing women aged 15–49 from utilizing VCT.

## Methods

### Data source

This study used secondary data collected by 2016 Ethiopian Demographic Health Survey (EDHS). EDHS 2016 is nationally representative and the most recent national survey on HIV testing. The Ethiopian Central Statistical Agency (ECSA) conducted the survey, with a technical assistance from Strategic consulting & communications for a digital world/ICF International. Data were collected according to a standard protocol of Demographic and Health Survey (DHS), which had three core survey questionnaires; the Household Questionnaire, the Woman’s Questionnaire and the Man’s Questionnaire. These questionnaires were translated into three major languages of the country— Amharigna, Afan Oromo, and Tigrigna. The questionnaires were pretested in all three languages before the start of fieldwork and the properly trained interviewers interviewed respondents. ICF International staff and representatives from other organizations participated in fieldwork monitoring. A quality control team was present in each of 9 regions and 2 city administrations [18].

### Study population and sampling procedure

**Fig. 1:**
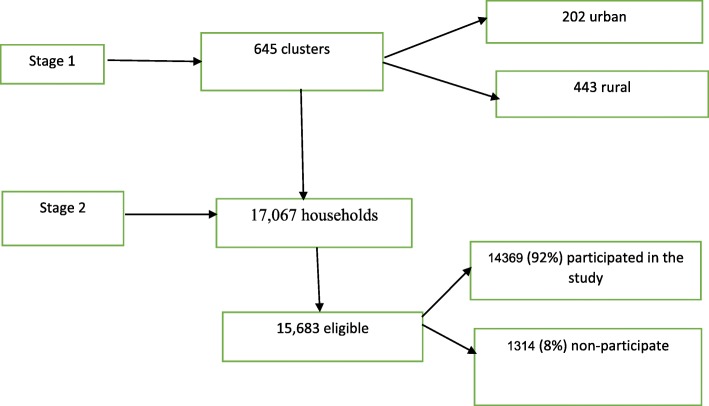
Sampling procedure and participation rate

The 2016 EDHS used a non-proportionate two-stage sampling design with stratification into urban and rural areas. At step one of the sampling, 645 clusters (202 urban and 443 rural) were selected on the basis of the 2007 Ethiopian population and housing census sampling frame [19]. The second phase of the sampling involved a complete listing of households in each selected cluster followed by a systematic random sampling of approximately 28 households from each cluster (giving a total of 18,008 households), of which 17,067 households were occupied and 14,369 out of 15,683 eligible participants were interviewed [19] (Fig. 1).

### Measurements

The dependent variable for the study was HIV testing and it was measured by asking the question: ‘Have you ever tested for HIV?’. The independent variables included socio-demographic characteristics: age, marital status, religion, residence, region, socio-economic position (SEP), HIV/AIDS-related knowledge, HIV/AIDS-related stigma towards people living with HIV/AIDS, and risky sexual behavior.

### Operational definitions

An index of the study participants’ socio-economic position was developed based on their educational level, wealth index, and occupational status. Primarily, wealth index and occupational status sub-variable were grouped into three categories and educational level sub-variable was grouped into four categories and then added together. This summation index gave a value ranging from 2 to 9. Participants’ SEP was categorized as low (score 2–5), middle (score 6–7), and high (score 8–9). Religion was categorized into five groups: Orthodox, Catholic, Protestant, Muslim and other. HIV/AIDS-related knowledge index was built from the answers to six questions: three questions on knowledge of HIV prevention and three on misconceptions about modes of HIV transmission. It was categorized as low (score ≤ 3), high (score 4–5), or comprehensive (score 6) knowledge. To measure participant’s risky sexual behavior history, participants were given five questions associated to their sexual behaviors (see Table 1) earlier to the survey. Having had any STI in last 12 months, having had genital sore/ulcer in last 12 months, having had genital discharge in last 12 months, having had at least one sexual partner other than husband in last 12 months, and having had multiple life time sexual partner were the questions asked to assess risky sexual behaviors of the participants. These were combined into an index of risky sexual behavior with three categories: “No risk” (score 0), “Some risk” (score 1) and “High risk” (score ≥ 2). According to the Joint United Nations Programme on HIV/AIDS (UNAIDS, 2014**)** definition, HIV-related stigma refers to negative beliefs, feelings and attitudes towards people living with HIV, their families and people who work with them.

**Table 1:** VCT uptake in relation to HIV/AIDS-related knowledge, HIV/AIDS-related stigma and risky sexual behavior history among Women in Ethiopia, 2016

Variables	Urban		Rural	
	*N* (%)	Ever HIV tested (%)	*n* (%)	Ever HIV tested (%)
Overall	5232 (36.4)	3802 (72.7)	9137 (63.6)	3800 (41.6)
Knowledge Indicator				
Reduce risk of getting HIV: always use condoms during sex (Yes)	4009 (76.6)	3066 (58.6)	4628 (50.7)	2356 (25.8)
Reduce risk of getting HIV: have 1 sex partner only, who has no other partners (Yes)	4108 (78.5)	3069 (58.7)	6041 (66.1)	2748 (30.1)
Can get HIV from mosquito bites (No)	3582 (68.5)	2660 (50.8)	4239 (46.4)	1972 (21.6)
Can get HIV by sharing food with person who has AIDS (No)	4848 (92.7)	3582 (68.5)	6630 (72.6)	3169 (34.7)
Can get HIV by witchcraft or supernatural means (No)	4773 (91.2)	3512 (67.1)	7011 (76.7)	3193 (34.9)
A healthy looking person can have HIV (Yes)	3855 (73.7)	2898 (55.4)	5368 (58.8)	2556 (28.0)
Stigma Indicator				
Would be ashamed if someone in the family had HIV (Disagree)	4180 (79.9)	3140 (60.0)	4389 (48)	2109 (23.1)
Would buy vegetables from vendor with HIV (Yes)	4106 (78.5)	3149 (60.2)	3199 (35.0)	1660 (18.2)
Children with HIV should be allowed to attend school with children without HIV (Yes)	4168 (79.7)	3182 (60.8)	3910 (42.8)	2046 (22.4)
People hesitate to take HIV test because reaction of other people if positive (No)	1376 (26.3)	963 (18.4)	2742 (30.0)	1018 (11.1)
People talk badly about people with or believed to have HIV (No)	2357 (45.0)	1771 (33.8)	3814 (41.7)	1661 (18.2)
People with or believed to have HIV lose respect from other people (No)	2598 (49.7)	1967 (37.6)	4044 (44.3)	1737 (19.0)
Risky Sexual Behavior Indicator				
Had any STI in last 12 months (Yes)	35 (0.7)	29 (0.6)	49 (0.5)	22 (0.2)
Had genital sore/ulcer in last 12 months (Yes)	74 (1.4)	63 (1.2)	133 (1.5)	59 (0.6)
Had genital discharge in last 12 months (Yes)	98 (1.9)	84 (1.6)	161 (1.8)	79 (0.9)
At least one sexual partner other than husband in last 12 months	362 (6.9)	964 (18.4)	106 (1.2)	861 (9.4)
Had multiple life time sexual partner (Yes)	1066 (20.4)	964 (18.4)	1579 (17.3)	861 (9.4)

Six questions that reflected negative attitudes about people living with HIV/AIDS were used to create a stigma index. This index was categorized as “No stigma” (score 6), “Low stigma” (score 4–5), “Moderate stigma” (score 2–3), and “High stigma” (score ≤ 1).

### Data analysis

**Fig. 2:**
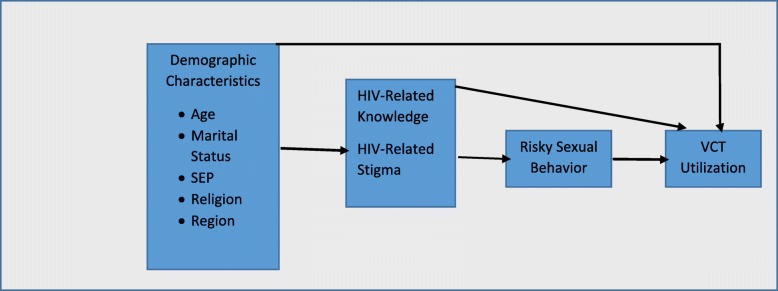
conceptual and analytical framework for studying determinants of VCT utilization by women (both urban and rural residents)

The data were analyzed using SPSS version 24. Logistic regression was used to assess the associations between the dependent and independent variables. In the regression models, information on age, marital status, SEP, religion, region, HIV/AIDS-related stigma, HIV/AIDS-related knowledge, and risky sexual behavior history, were included as independent variables (see framework in Fig. 2).

The association between the dependent and each independent variable was analyzed using logistic regression analysis. A stepwise approach was employed in building the multivariate models. In the first model, the association between the socio-demographic variables and VCT was assessed. In the second model, both sociodemographic characteristics and more proximal determinants (HIV/AIDS-related knowledge and stigmatizing attitudes) were included. In the final model, all variables were included. The associations were presented as odds ratios (OR) and 95% confidence intervals. The analysis was stratified by residence (urban and rural) due to interaction.

## Results

### Back ground characteristics and HIV testing history

The mean age of the participants was 28 years and 59.4% in urban and 79.4% in rural were married. Fifty two percent of women in urban were at middle socio economic position, but the majority of rural participants (83.1%) were at lower socio economic position. Eighty percent of the study participants reported that they knew a place where they could get tested for HIV. Seventy three percent of urban and 41.6% of rural women had ever tested for HIV. Overall prevalence of ever having tested for HIV among women aged 15–49 was 53% (95% CI, 52,45) and 93.1% of them who had tested had received result from last HIV test (Tables 1 and 2).

**Table 2:** Frequency distribution of VCT uptake among Women in Ethiopia in 2016 by urban–rural residence

Variables	Urban			Rural		
	*N* (%)	Ever HIV tested (%)	*P*-value	*N* (%)	Ever HIV tested (%)	*P*-value
Age in 5-year groups						
15–19	1216 (23.2)	13.3	< 0.001	1996 (21.8)	13.9	< 0.001
20–24	1080 (20.6)	21.4		1634 (17.9)	21.4	
25–29	982 (18.8)	22.5		1629 (17.8)	20.6	
30–34	725 (13.9)	16.9		1316 (14.4)	16.3	
35–39	572 (10.9)	12.5		1159 (12.7)	13.1	
40–44	379 (7.2)	7.8		804 (8.8)	9.3	
45–49	278 (5.3)	5.5		599 (6.6)	5.5	
Marital Status						
Never Married	2125 (40.6)	30.2	< 0.001	1886 (20.6)	12.3	< 0.001
Ever Married	3107 (59.4)	69.8		7251 (79.4)	87.7	
Socio Economic Position						
Low	1699 (32.5)	28.9	< 0.001	8056 (88.2)	83.1	< 0.001
Middle	2728 (52.1)	52.4		985 (10.8)	14.8	
High	805 (15.4)	18.6		96 (1.1)	2.1	
Religion						
Orthodox	2989 (57.1)	61.1	< 0.001	3238 (35.4)	44.5	< 0.001
Catholic	29 (0.6)	0.6		55 (0.6)	0.5	
Protestant	678 (13)	12.5		1933 (21.2)	20.0	
Muslim	1520 (29.1)	25.5		3820 (41.8)	34.4	
Others	16 (0.3)	0.3		91 (1)	0.6	
Region						
Tigray	417 (8)	8.8	< 0.001	1239 (13.6)	20.0	< 0.001
Afar	191 (3.7)	3.9		831 (9.1)	5.9	
Amhara	246 (4.7)	5.2		1408 (15.4)	18.2	
Oromia	249 (4.8)	4.2		1426 (15.6)	11.9	
Somali	271 (5.2)	2.8		692 (7.6)	3.0	
Benishangul	145 (2.8)	3.1		864 (9.5)	9.9	
SNNPR	225 (4.3)	4.1		1547 (16.9)	16.8	
Gambela	339 (6.5)	6.6		555 (6.1)	7.9	
Harari	511 (9.8)	10.4		352 (3.9)	3.7	
Addis Adaba	1815 (34.7)	34.9		0 (0)	0.0	
Dire Dawa	823 (15.7)	16.0		223 (2.4)	2.8	
Knowledge Indicator						
Low Knowledge	4488 (85.8)	85.8	0.804	7362 (80.6)	80.7	0.003
High Knowledge	725 (13.9)	13.8		1649 (18.0)	18.4	
Comprehensive Knowledge	19 (0.4)	0.4		126 (1.4)	0.9	
Stigma Indicator						
High Stigma	381 (7.3)	4.8	< 0.001	3037 (33.2)	26.6	< 0.001
Moderate Stigma	2182 (41.7)	40.7		3584 (39.2)	40.9	
Low Stigma	2246 (42.9)	45.7		2156 (23.6)	27.2	
No Stigma	423 (8.1)	8.7		360 (3.9)	5.3	
Sexual Risky Behavior Indicator						
High Risk	294 (5.6)	6.9	< 0.001	191 (2.1)	2.7	< 0.001
Some Risk	1020 (19.5)	23.9		1631 (17.9)	22.9	
No Risk	3918 (74.9)	69.2		7315 (80.1)	74.3	

### HIV/AIDS-related knowledge

The participant’s knowledge on all specific measured parameters was higher among urban than among rural women both in terms of HIV prevention and means of transmission. About half of the participants in rural and 76.6% in urban knew that regular condom usage during sex reduce risk of getting HIV. Eighty six percent of women in urban and 80.6% of rural women were in low knowledge indicator category. Similarly, 53.6% rural and 31.5% urban women residents responded they still believe that they might get infected with HIV by mosquito bite and 41.2% of women living in rural didn’t know the fact that healthy looking person can have HIV (Tables 1 and 2).

### HIV/AIDS-related stigma

Fear of loss of respect and others reaction if tested positive were measured parameters that indicated high stigma level among both urban and rural participants. The majority of rural participants said: children with HIV should not be allowed to attend school with children without HIV, they would be ashamed if someone in the family had HIV and they would not buy vegetables from vendor with HIV. Only 11.1% rural and 18.4% urban women who had fear of other’s reaction if tested positive, had ever tested for HIV. Generally, women in rural had more stigmatizing attitude toward people living with HIV/AIDS (PLWHA) than those live in urban (Table 1). Eighteen point 6 % (18.6%) urban and 2.1% rural women who had high stigmatizing attitude toward people living with HIV/AIDS (PLWHA) had been ever tested (Tables 2 & 3).

**Table 3:** Logistic regression analysis of determinants of VCT utilization among urban Women in Ethiopia, 2016

Variables	Ever been tested for HIV		
	Model 1 OR(95% CI)^a^	Model 2 OR(95% CI)^b^	Full Model OR(95% CI)^c^
Age (Ref: 15–17, 19, 20)			
20–24	**2.5 (2.04–3.06)**	**2.51 (2.05–3.08)**	**2.31 (1.88–2.83)**
25–29	**4.24 (3.29–5.47)**	**4.35 (3.36–5.62)**	**3.92 (3.02–5.07)**
30–34	**4.47 (3.31–6.03)**	**4.41 (3.26–5.98)**	**3.79 (2.78–5.15)**
35–39	**2.74 (2.03–3.68)**	**2.66 (1.97–3.59)**	**2.3 (1.7–3.13)**
40–44	**1.72 (1.25–2.39)**	**1.65 (1.19–2.29)**	1.36 (0.97–1.9)
45–49	1.39 (0.98–1.98)	1.33 (0.93–1.9)	1.12 (0.78–1.61)
Marital Status (Ref: Never Married)			
Ever Married	**4.81 (3.95–5.85)**	**5.01 (4.11–6.12)**	**4.79 (3.92–5.86)**
Socioeconomic Position (Ref: Lower)			
Middle	**2.14 (1.81–2.53)**	**2 (1.69–2.37)**	**1.94 (1.64–2.3)**
High	**3.79 (2.89–4.98)**	**3.42 (2.6–4.5)**	**3.32 (2.52–4.38)**
Religion (Ref: Orthodox)			
Catholic	0.64 (0.23–1.77)	0.73 (0.27–2.02)	0.79 (0.28–2.2)
Protestant	**0.75 (0.59–0.96)**	0.8 (0.63–1.03)	0.85 (0.66–1.09)
Muslim	**0.63 (0.52–0.76)**	**0.64 (0.53–0.77)**	**0.68 (0.56–0.83)**
Others	**0.28 (0.09–0.85)**	0.34 (0.11–1.04)	0.39 (0.13–1.2)
Region (Ref: Tigray)			
Afar	1.02 (0.63–1.65)	0.98 (0.6–1.59)	0.96 (0.59–1.57)
Amhara	0.89 (0.57–1.39)	0.82 (0.52–1.29)	0.82 (0.52–1.29)
Oromia	**0.38 (0.25–0.57)**	**0.36 (0.23–0.54)**	**0.38 (0.25–0.57)**
Somali	**0.18 (0.12–0.27)**	**0.23 (0.15–0.35)**	**0.24 (0.16–0.37)**
Benishangul	1.2 (0.7–2.07)	1.09 (0.63–1.88)	1.14 (0.66–1.98)
SNNPR	0.69 (0.44–1.09)	0.71 (0.45–1.13)	0.72 (0.45–1.15)
Gambela	0.68 (0.45–1.03)	**0.65 (0.43–0.99)**	**0.61 (0.4–0.94)**
Harari	1.01 (0.70–1.46)	0.94 (0.65–1.37)	1.01 (0.69–1.47)
Addis Adaba	0.79 (0.58–1.06)	0.75 (0.56–1.01)	0.76 (0.56–1.02)
Dire Dawa	0.99 (0.70–1.38)	0.86 (0.61–1.21)	0.88 (0.62–1.25)
HIV - Related Comprehensive Knowledge Index (Ref:Low)			
High Knowledge		1.11 (0.9–1.36)	1.08 (0.88–1.33)
Comprehensive Knowledge		2.74 (0.82–9.15)	2.8 (0.84–9.34)
Stigma Indicator (Ref:No Stigma)			
High Stigma		**0.35 (0.24–0.51)**	**0.36 (0.25–0.52)**
Moderate Stigma		0.82 (0.61–1.09)	0.83 (0.62–1.11)
Low Stigma		1.04 (0.78–1.39)	1.05 (0.79–1.41)
Sexual Risky Behaviour Indicator (Ref: No Risk)			
High Risk			**2.56 (1.7–3.86)**
Some Risk			**2.06 (1.64–2.6)**

Boldface entries were used to show 95% CI of the ratio doesn't contain 1, *p*-value is less than 0.05 and there are significant associations between the variables

### Risky sexual behavior history

Having had multiple sexual partners was one of the most common risky sexual behavior observed among participants. Eighteen point 4 % (18.4%) urban and 9.4% rural participants who had had multiple sexual partners had ever been tested (Table 1). Only 6.9% of women in urban and 2.7% of rural women with high risky sexual behaviors had ever tested for HIV (Table 2).

### Factors associated with VCT utilization

In the logistic regression analysis, utilization of VCT among women was positively associated with marital status (OR 4.79, 95% CI: 3.92–5.86) in urban and (OR 3.46, 95% CI: 2.93–4.1) in rural. Similarly positive association was found between VCT utilization and socio-economic positions (SEP) (OR 8.21, 95%: 4.63–14.56) in rural (OR 3.32, 95% CI: 2.52–4.38) in urban areas. Compared with 15–19 years of age, age less than 40 years in urban and less than 35 years in rural was strongly and positively associated (OR 2.3, 95% CI: 1.7–3.13), (OR 1.43, 95% CI: 1.19–1.73) respectively with VCT use. However, age > 45 and VCT utilization in rural area was found to be negatively associated (OR 0.71, 95% CI: 0.57–0.9) compared with 15–19 years of age. Being Muslim in urban and protestant in rural areas were found to be strongly and negatively associated (OR 0.68, 95% CI: 0.56–0.83**),** (OR 0.73, 95% CI:0.16–0.86) with VCT utilization respectively. Negative associations was also found between VCT uptake in rural parts of different regions in the country (Tables 3 and 4). Similarly negative association (OR 0.36, 95% CI: 0.25–0.52) in urban and (OR 0.4, 95% CI:0.31–0.51) in rural was found between stigma and VCT uptake. Risky sexual behaviors was positively associated (OR 2.56, 95% CI: 1.7–3.86) in urban and (OR 1.62, 95% CI: 1.18–2.22) in rural with VCT uptake.

**Table 4:** Logistic regression analysis of determinants of VCT utilization among rural Women in Ethiopia, 2016

Variables	Ever been tested for HIV		
	Model 1 OR(95% CI)^a^	Model 2 OR(95% CI)^b^	Full Model OR(95% CI)^c^
Age (Ref: 15–17, 19, 20)			
20–24	**1.75 (1.48–2.07)**	**1.75 (1.47–2.07)**	**1.72 (1.45–2.04)**
25–29	**1.54 (1.29–1.84)**	**1.52 (1.28–1.82)**	**1.49 (1.24–1.78)**
30–34	**1.47 (1.22–1.77)**	**1.5 (1.25–1.81)**	**1.43 (1.19–1.73)**
35–39	1.14 (0.94–1.38)	1.15 (0.95–1.4)	1.09 (0.89–1.32)
40–44	1.18 (0.96–1.45)	1.19 (0.97–1.47)	1.11 (0.9–1.37)
45–49	**0.78 (0.62–0.98)**	**0.78 (0.62–0.98)**	**0.71 (0.57–0.9)**
Marital Status (Ref: Never Married)			
Ever Married	**3.3 (2.79–3.89)**	**3.58 (3.03–4.23)**	**3.46 (2.93–4.1)**
Socioeconomic Position (Ref: Lower)			
Middle	**2.71 (2.32–3.17)**	**2.52 (2.16–2.95)**	**2.53 (2.16–2.96)**
High	**9.5 (5.37–16.81)**	**8.34 (4.7–14.79)**	**8.21 (4.63–14.56)**
Religion (Ref: Orthodox)			
Catholic	0.6 (0.33–1.1)	0.62 (0.34–1.14)	0.64 (0.35–1.16)
Protestant	**0.69 (0.58–0.82)**	**0.71 (0.6–0.84)**	**0.73 (0.61–0.86)**
Muslim	1.07 (0.92–1.25)	1.06 (0.92–1.24)	1.08 (0.93–1.26)
Others	**0.39 (0.24–0.64)**	**0.45 (0.27–0.74)**	**0.46 (0.28–0.77)**
Region (Ref: Tigray)			
Afar	**0.18 (0.14–0.24)**	**0.18 (0.14–0.23)**	**0.19 (0.15–0.24)**
Amhara	**0.54 (0.45–0.63)**	**0.48 (0.4–0.57)**	**0.46 (0.39–0.55)**
Oromia	**0.26 (0.21–0.32)**	**0.23 (0.19–0.29)**	**0.24 (0.2–0.29)**
Somali	**0.1 (0.07–0.13)**	**0.11 (0.08–0.14)**	**0.11 (0.08–0.15)**
Benishangul	**0.43 (0.35–0.53)**	**0.37 (0.29–0.45)**	**0.37 (0.3–0.46)**
SNNPR	**0.55 (0.45–0.68)**	**0.53 (0.43–0.65)**	**0.55 (0.44–0.67)**
Gambela	0.86 (0.67–1.11)	**0.7 (0.55–0.91)**	**0.69 (0.54–0.89)**
Harari	**0.3 (0.22–0.4)**	**0.28 (0.21–0.37)**	**0.29 (0.21–0.39)**
Dire Dawa	**0.48 (0.34–0.67)**	**0.39 (0.28–0.55)**	**0.41 (0.29–0.58)**
HIV - Related Comprehensive Knowledge Index (Ref:Low)			
High Knowledge		0.98 (0.87–1.11)	0.98 (0.87–1.1)
Comprehensive Knowledge		**0.59 (0.39–0.9)**	**0.59 (0.39–0.89)**
Stigma Indicator (Ref:No Stigma)			
High Stigma		**0.4 (0.32–0.51)**	**0.4 (0.31–0.51)**
Moderate Stigma		**0.6 (0.47–0.76)**	**0.59 (0.47–0.75)**
Low Stigma		**0.73 (0.57–0.93)**	**0.72 (0.56–0.91)**
Sexual Risky Behavior Indicator (Ref: No Risk)			
High Risk			**1.62 (1.18–2.22)**
Some Risk			**1.35 (1.19–1.53)**

Boldface entries were used to show 95% CI of the ratio doesn't contain 1, *p*-value is less than 0.05 and there are significant associations between the variables

In model 1 where the association between the socio-demographic variables and VCT was assessed, age between 20 and 44, being married and having higher SEP (≥ middle) were associated with greater VCT use than being younger, never married and having lower SEP, respectively. Being Muslim in urban, protestant and older age (> 45) in rural were the socio-demographic factors that were significantly and negatively associated with VCT utilization compared with orthodox and younger age respectively (Tables 3 and 4).

In model 2, both sociodemographic characteristics and more proximal determinants, HIV/AIDS-related knowledge and stigmatizing attitudes, were included into analysis. In this model, having stigmatizing attitude, having comprehensive knowledge and being protestant in rural stratum were negatively associated with VCT use compared with no stigma, low knowledge index and being orthodox respectively. Better SEP (≥ middle) and being married were positively associated with VCT utilization compared with lower SEP and being never married (Tables 3 and 4). In the full model at which all variables were included into analysis and risky sexual behavior was found to be an important predictor of VCT utilization. Being ever married, better SEP, having history of risky sexual behavior and age between 20 and 39 were found to be factors associated positively with VCT compared with those never married, lower SEP, no risky sexual behaviors and ages 15–19 respectively in the full model. Having stigmatizing attitude in both urban and rural and having comprehensive knowledge in rural area were negatively associated VCT in rural stratum in the full model (Tables 3 and 4).

## Discussion

VCT service uptake among Ethiopian women (aged 15–49 years) in this study is 52.9% and wide range of difference was observed between women in urban and rural at VCT utilization. Age, marital status, socio economic positions, religion, regions, stigma, and risky sexual behaviors are important variables identified for VCT service uptake. Compared with other studies conducted in Ethiopia among men before 13 years and in 13 sub-Saharan Africa countries as well as in rural Tanzania on uptake of HIV voluntary counselling and testing services, the prevalence of being tested for HIV in current study is higher [16, 18, 20, 21]. This is due to efforts made in Ethiopia in the last years to integrate and expand VCT service. The initiation of affordable and effective medical care for people living with HIV/ADIS (PLWHA) might have also increased the demand of individuals for HIV testing. More importantly, the promotion of voluntary counseling and testing (VCT) by the government in the las two decades as one of the key policy responses to the HIV/AIDS epidemic, both as a primary prevention strategy and an entry point to other HIV/AIDS related services in the country has played a positive role for such improvement. However, the estimated prevalence of ever being tested for HIV in this study is still comparable with reports from four African countries (Nigeria, Mozambiqu, Kenya and Burkina Faso) [22–26].

Marriage is positively associated with VCT utilization in this study. Married women were found to utilize VCT better than those unmarried. This is consistent with the study done on factors affecting utilization of Voluntary HIV Counseling and Testing Services in Northwest Ethiopia [27]. This may probably be due to perceived personal risk about being infected with HIV in their past or current relationships.

HIV/AIDS related stigma and VCT uptake among women in Ethiopia was negatively and strongly associated in our study (OR 0.36, 95% CI: 0.25–0.52, in urban and OR 0.4, 95% CI:0.31–0.51, in rural). HIV/AIDS is the most stigmatized disease than any other health conditions and people living with HIV or the social groups to which they belong to have been suffering from stigmatization since the beginning of HIV epidemic. People living with HIV/AIDS (PLWHA) face not only personal medical problems but also social problems associated with the disease [28–30]. Therefore, due to fear of stigmatization women would not be willing to use VCT services. Many studies indicated that biomedical HIV prevention strategies, is predicated based on regular HIV testing [31–33]. However, anticipated stigma remains significant predictors of HIV testing behaviors hindering many people especially women from being tested and disclose their HIV status. Fear of stigma limits the efficacy of HIV-testing programs across sub-Saharan Africa, because in most villages everyone knows eventually who visited test sites [34–37]. The notions in the community that associate HIV with socially, morally and religiously unaccepted behavior [38], might also contributed for stigmatizing attitude and ultimately impose negative impact on VCT utilization among women in Ethiopia.

Consistent with the studies conducted in UK on migrants from five sub-Saharan African communities (Congo, Kenya, Uganda, Zambia, Zimbabwe) [39] and Burkina Faso [40], those who had history of high risky sexual behavior were more likely to utilize VCT in this study. Individuals with risky sexual behaviors lives under persistent fear and uncertainty about their own sero-status. They are usually suspicious and worries that they might have infected with HIV. This could have urged them to develop habits of seeking VCT service than individuals with less or no risky sexual behavior. Several other studies also reported increased HIV testing practice among individuals with risky sexual behaviors [41–43].

VCT utilization also significantly associates with religion in this study. One explanation is the dual role (positive and negative) religious view and practice might have when it comes to HIV/AIDS. Religious views could be challenges to communicate or educate about sexual health in religious communities and may become obstacle or barrier to VCT as well as other HIV preventions efforts (negative role). At the same time it might provide PLWHA spiritual well-being and social support (positive role), that could serve as facilitators to HIV treatment and care [44].

Muslim women in urban showed a low rate for VCT utilization than those from other religious and cultural backgrounds in this study and it’s consistent with studies done in Ghana and Ethiopia [16, 45]. In Muslim cultures, sexuality is considered as private matter, a taboo topic for discussion and the social stigma attached to HIV/AIDS, that also exists in all societies is much more pronounced which prevents those at risk from coming forward for appropriate counseling, testing and treatment [46]. Social norms and ideas in Muslim communities, which associate HIV/AIDS with homosexuality, extramarital sex and drug use might have negatively affected VCT use among women [38]. Consistent with our study, being Muslim was found to be inversely associated with utilization of VCT in a cross-sectional survey conducted among men in Ethiopia [16]. Muslim women are less likely to use VCT services probably because of restricted role of women in Muslim community and therefore limiting their decisions to use VCT services.

In addition to religious factors, health extension program that has dramatically improved access to health and HIV prevention service in Ethiopia has stared recently in urban compared to rural area. This could be addition reason for low utilization of VCT service among urban Muslim women.

Protestant women in rural also showed low VCT uptake in this study. In Protestant community, there is a notions that HIV/AIDS comes due to promiscuity, homosexuality or as punishment from God for the sin (adultery) he/she did, contribute for such outcomes.

Women who ever married were significantly more likely to be tested for HIV than those who were never married in our study. This could result from compulsory counselling and testing promotion for couples intending to get married by different organizations including UNAIDS and religious groups. The perinatal HIV testing service available in most health institutions, might also contributed for high rate of testing among married women. Ethiopia Federal ministry of Health also encourages couples to learn about their HIV status and make informed decisions about their future that might have played positive role for married women to better utilize VCT. This suggest that the government and health organizations could play more positive role for all women not only for those going to marry, to better utilize VCT through such kinds of counselling and testing promotions. Similar positive association was reported in separate studies conducted in Kenya, Tanzania and Ethiopia [47–49].

Socio-economic position (SEP) was another important factor for VCT utilization among women in this study. An index of participants’ socio-economic position was developed on the bases of their educational level, wealth index, and occupational status. Participants’ in the middle and high SEP categories were more likely to be tested than those in low SEP. SEP encompasses not just income but also educational level, employment (financial security). SEP affect utilization of health services in many aspects including VCT service uptake, even in countries with universal health care system [50, 51]. Women at higher socio-economic positions have better educational level and economically privileged to seek and access health service, including VCT services than those at lower socio-economic position. Hence, the government needs to make persistent advance in popular education, in increasing the individual income and decrease unemployment rate.

The cross-sectional nature of the data limits ability to draw casual inferences and using secondary source data like this is subjected to missing of important information (Variables) and recall bias are among the limitations of this study.

## Conclusion

With the development of affordable and effective medical care for people living with HIV, the demand for HIV counselling and testing among women has rapidly increased in Ethiopia. However, in this study we found that stigmatizing attitude remains major factor, which is negatively affecting VCT uptake among women in Ethiopia. Concerted efforts should be made by all stakeholders to mitigate stigma, improve socio economic inequities and increase awareness on the benefit of VCT in controlling HIV in the society. In this aspect, the role of religious leader, schools, health extension workers and community leaders should not be undermined. Implementation and strengthening of alternative VCT services that include mobile VCT, routine offer of VCT at workplace, schools, religious congregations and home-based VCT service is highly recommended to increase uptake of VCT.

## Data Availability

The data is available on https://dhsprogram.com/Data/. To request dataset access, one must first register as a DHS data user on DHS website. Dataset access is only granted for legitimate research purposes. Dataset requests must include contact information, a research project title, and a description of the analysis one propose to perform with the data.

## Abbreviations

AIDS: Acquired Immune Deficiency Syndrome
CDC: Center for Disease Control and Prevention
DHS: The Demographic and Health Surveys
ECSA: The Ethiopian Central Statistical Agency
EDHS: Ethiopia Demographic and Health Survey
ICF: Strategic consulting & communications for a digital world
PLWHA: People Living With HIV/AIDS
SEP: Socio-economic Position
SPSS: Statistical Package for the Social Sciences
STI: Sexually Transmitted Infections
UNAIDS: The joint United Nations program on HIV/AIDS
VCT: Voluntary Counselling and Testing
